# Intrahepatic cholestasis of pregnancy: an evaluation of obstetric management in German maternity units

**DOI:** 10.1007/s00404-022-06754-3

**Published:** 2022-08-28

**Authors:** Leonie Zehner, Maria Mai, Anna M. Dückelmann, Amr Hamza, Christel Eckmann-Scholz, Nicolai Maass, Ulrich Pecks

**Affiliations:** 1 Medical Faculty, Christian-Albrechts-Universität Kiel, Christian-Albrechts-Platz 4, 24118 Kiel, Germany; 2 Department of Gynecology and Obstetrics, Charité – Universitätsmedizin Berlin, Augustenburger Platz 1, 13353 Berlin, Germany; 3 Department of Gynecology and Prenatal Medicine, Kantonspital Baden, Im Ergel 1, 5400 Baden, Germany; 4 Department of Gynecology, Obstetrics and Reproductive Medicine, Kirrberger Straße, 66424 Homburg, Germany; 5 Department of Gynaecology and Obstetrics, Universitätsklinikum Schleswig-Holstein, Arnold-Heller-Straße 3, 24105 Kiel, Germany

**Keywords:** Bile acids, Delivery timing, Guidelines, Management

## Abstract

**Purpose:**

Intrahepatic cholestasis of pregnancy (ICP) is associated with adverse fetal and neonatal outcome. Evidence for improvement by obstetric management is sparse. Common international guidelines recommend induction of labor before term, however, they differ in recommendations of monitoring the disease and time point of active management. So far, an official guideline for treatment and management of ICP in Germany does not exist. This study aims to compile common practice and policy in obstetric management of ICP in German maternity units. The objective is to gather obstetricians’ opinion on management of ICP, and to estimate the need for standardization of current practice in Germany on the background of existing evidence.

**Methods:**

A questionnaire focusing on indications for interventions was developed including fourteen multiple-choice questions comprising the areas of diagnostic criteria, laboratory testing, fetal monitoring, treatment, and delivery timing. The survey was sent to 699 maternity clinics and was distributed to participants of the annual congress hosted by the German society of perinatal medicine (DGPM). Collected data were summarized and evaluated in relation to available evidence and existing guidelines. Descriptive statistics and Fisher's exact test were used.

**Results:**

334 completed questionnaires returned corresponding to a response rate of 48.1%. Coinciding with existing international guidelines, 48.8% of the participants acknowledge bile acid concentrations above 10 µmol/L to be indicative of ICP. 85.0% of obstetricians recommend antenatal testing with cardiotocography, exceeding common standards of maternity policy guidelines; 50.3% execute active management in ICP-affected pregnancies as they generally recommend a delivery between 37 + 0 and 38 + 6 weeks of gestation. Although recent studies evinced a risk of stillbirth in ICP-affected pregnancies not until a bile acid concentration of > 100 µmol/L, 22.2% of the respondents recommend delivery before 37 + 0 weeks of gestation due to raised bile acids of 40–99 µmol/L.

**Conclusions:**

Opinions on the management of ICP in German maternity units differ widely and partly deviate by large from international standards. Reasons for this may be the lack of a national guideline and the low awareness due to the rarity of the disease on the one hand and the very slow dynamics in evidence generation and thus the uncertainty about the actual risks and optimal management on the other. The present data highlight the need for further research and clinical guidelines to standardize and optimize treatment based on the best available evidence.

**Supplementary Information:**

The online version contains supplementary material available at 10.1007/s00404-022-06754-3.

## What does this study add to the clinical work

**Table:** 

At the time of the study a diverse picture and a lack of clarity existed regarding the management of ICP in German maternity units. In order to standardize German practices and educate physicians to improve the treatment of ICP the Working Group on Obstetrics and Prenatal Medicine—Section on Maternal Disorders (AGG) of the German Society of Obstetrics and Gynecology has recently developed recommendations.	

## Introduction

Intrahepatic cholestasis of pregnancy (ICP) is a rare condition in pregnancy affecting about 1% of women. It is the most common pregnancy-specific liver disorder [1]. Mostly, it develops during the late second and third trimester [2]. Main features and diagnostic criteria are pruritus associated with elevated serum bile acid levels (above 10 µmol/L fasting, and 15 µmol/L non-fasting value) and/or disturbed liver function tests (LFT) [3]. Yet, ICP is diagnosed by exclusion and confirmed by the postpartal regression of symptoms and laboratory parameters [4]. Although not expected to cause serious maternal complications, it is associated with adverse perinatal outcomes, including spontaneous preterm birth (30–40%), meconium stained amniotic fluid (16–58%), neonatal respiratory distress syndrome (29%), and, most critically, sudden fetal death with the highest risk of occurrence after 37 weeks of gestation [5]. The diagnostic criteria for ICP have changed over time and vary from report to report, making it difficult to estimate actual complication rates [6]. For most women diagnosed with ICP, management is targeted toward both symptomatic relief and prevention of stillbirth [7]. However, evidence for optimized management of ICP is scarce. The lack of guidelines with consensual diagnostic and therapeutic recommendations hinders the implementation of a uniform clinical practice in Germany. Even on an international level a consensus is missing. Bicocca et al. reviewed six available national guidelines on ICP and concluded that the recommendations vary and are inconsistent [8]. A major difference among guidelines resides in the indication of induction of labor (IOL) on a given week based on a given ICP severity as well the pharmacological management of ICP [8–10]. The European guidelines recommend Ursodeoxycholic acid (UDCA) as a first-line treatment to alleviate maternal symptoms [11]. Whether UDCA has beneficial fetal effects is a matter of debate [12, 13]. The indication and dosage of UDCA remain a subject of debate [14]. This survey aims to provide an overview of the current practice of obstetric management in German maternity units in the light of controversial guidelines and the relevant risk of stillbirth in ICP. A special highlight was given to investigate ICP management and the recommendations for IOL in the late third trimester. Moreover, we wanted to obtain information regarding diagnostic criteria, laboratory tests, prenatal monitoring, medical treatment, counseling practices, and postpartum monitoring in German hospitals.

## Materials and methods

### Study and questionnaire design

We developed a questionnaire based on the current published literature, clinical experience, and existing international guidelines on the management of ICP. We composed it in fourteen multiple-choice questions, including questions about diagnostic criteria, the consultation process, and clinical practice related to laboratory testing, fetal monitoring, and medical management with UDCA. Depending on described circumstances of laboratory chemistry data at defined weeks of gestation, we asked the participant to provide their recommendations for active IOL or expective management. Finally, we asked the participants to recommend pre- and postpartum surveillance, including testing frequency. The questionnaire is available as supplementary material (Supplementary material 1).

### Study population

The study population included of a sample of gynecologists and obstetricians actively involved in clinical care. We sent the survey by postal letter with a prepared response envelope to the heads of obstetrical departments of the registered 315 perinatal care centers in Germany in September 2019. In this manuscript, Level III perinatal centers are referred to as centers with the highest level of specialization. Postal addresses were obtained from the Institute for Quality Assurance and Transparency (IQTIG). A reminder letter was sent in May 2020. In addition, we contacted 384 non perinatal center hospitals. Finally, we asked medical doctors in the field of obstetrics and maternofetal medicine during the congress of German Society of Perinatal Medicine in November 2019 in Berlin to participate in the survey. The medical doctors at the congress were asked in person thereby one hospital could have been represented by several participants. It can be assumed though that the evaluation of these questionnaires differ nevertheless. Even if hospitals had a written concept for the management of ICP, the participants of the congress were not at their working places, so had to fill out the answers to the best of their knowledge. All surveys were conducted anonymously.

### Statistical analysis

Data are presented descriptively, providing frequencies and percentages of answers given. Statistical analysis was performed using Excel for Microsoft Office 365 (2016, Microsoft Corporation) and Prism Version 8– GraphPad. Statistical significance of contingency tables was investigated with Fisher's exact test. Given answers were compared to demographic and professional characteristic. A *p *value of < 0.05 was considered statistically significant. Figures were generated by Tableau Version 2020.3 (Tableau software, Seattle, USA). Unanswered questions were defined as abstention. If respondents chose more than one multiple-choice option from the panel and only one answer had to be chosen, the answers were defined as indistinct.

## Results

147 out of 315 (46.7%) perinatal care centers, and 67 out of 379 (17.7%) maternity units completed our survey. Five surveys were returned because the obstetrics department has since closed, yielding an overall response rate of 30.8%. In addition, another 120 questionnaires were handed out and collected at the congress, so that a total of 334 questionnaires could be evaluated.

### Personal and demographic characteristics of respondents

The respondents were distributed across all federal states of Germany. The majority of respondents (63.5%) were females. 41.3% of the participants were specialized in obstetrics and perinatal medicine. Most participants indicated to be senior physicians (41.9%), senior consultant physicians (17.7%) or heads of the department (20.1%). 44.3% participants worked at perinatal care centers with highest specialty (level III) including university medical centers (8.1%). 10.5% of responding hospitals declared having more than 3000 births per year, while the majority indicated less than 2000 birth per year (68.3%). More detailed data are presented in Table 1. The minority of participants declared having written internal guidelines on the management of ICP available in their hospitals: 45.7% of them work in perinatal care centers with the highest specialty (level III) whereas 32.5% work in maternity hospitals.

**Table 1:** Personal and demographic characteristics of respondents

	*N* (%)
Sex	
Male	113 (33.8%)
Female	212 (63.5%)
Medical specialization	
Trainees in obstetrics and gynecology	29 (8.7%)
Obstetrics and gynecology	143 (42.8%)
Obstetrics and perinatal medicine	138 (41.3%)
Status of employment	
Resident physician	28 (8.4%)
Consultant physician	31 (9.3%)
Senior physician	140 (41.9%)
Senior consultant physician	59 (17.7%)
Head of department	67 (20.1%)
Working place	
Maternity clinic	92 (27.5%)
Perinatal care center level I	44 (13.2%)
Perinatal care center level II	43 (12.9%)
Perinatal care center level III	148 (44.3%)
Birth per year	
< 1000	77 (23.1%)
1000–1999	151 (45.2%)
2000–3000	63 (18.9%)
> 3000	35 (10.5%)

Data are *n* (% of respondents)

### Diagnostic criteria

95.5% and 79.3% considered that raised serum bile acids and generalized or localized (palms and/or soles) pruritus should be fulfilled mandatorily to diagnose ICP, respectively. 33.8% regarded an abnormal LFT (e. g. increase in aspartate transaminases activity) as essential for diagnosis. According to half of participants (48.8%) cut-off values for bile acids concentrations above ( >) 10 µmol/L should be used for definition. Yet, 65.3% considered reassessing the fasting bile acid status. Only 4.5% excluded a significant role of serum bile acid concentrations in the diagnostic algorithm (Table 2).

**Table 2:** Clinical features need for diagnosis of ICP

	*N* (%)
Pruritus	265 (79.3%)
Jaundice	15 (4.5%)
Bilirubin $\uparrow$	44 (13.2%)
Transaminases $\uparrow$	113 (33.8%)
γGT $\uparrow$	19 (5.7%)
Bile acids	319 (95.5%)
Bile acids > 10 µmol/L	163 (48.8%)
Bile acids > 15 µmol/L	58 (17.4%)
Bile acids > 40 µmol/L	63 (18.9%)
Bile acids > 100 µmol/L	6 (1.8%)
Missing value/Abstention	30 (9.0%)

Data are *n* (% of respondents)

### Laboratory testing

Once ICP is diagnosed recommendations for further laboratory testing varied greatly. As a matter of routine 0.9% recommended repeating laboratory tests daily. 6.0, 14.4, 33.2 and 11.1% tested every 2 to 3, 4 to 6, 7 to 13 and 14 or more days, respectively. 20.1% do not recommend any laboratory testing and 14.4% of the surveys could not be evaluated due to abstention or unclear information.

### Fetal monitoring

**Fig. 1:**
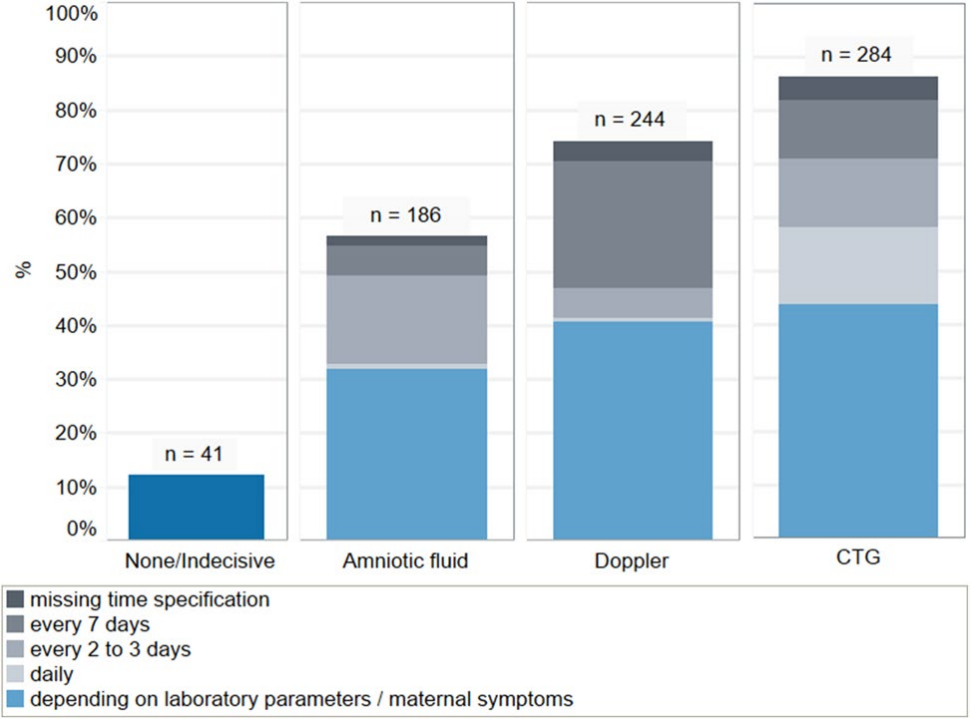
Recommended parameters of antenatal fetal surveillance and frequencies of obstetric measures

Most of the maternity clinics have adopted fetal monitoring policies. The recommended parameters of antenatal fetal surveillance used were Cardiotocogramm (*n* = 284; 85.0%), Doppler blood flow measurements (*n* = 244; 73.1%) and the determination of amniotic fluid volumes (*n* = 186; 55.7%). The frequencies of specified time periods varied greatly (Fig. 1). Only 10.8% reported that fetal monitoring is not necessary throughout the pregnancy.

### Medical treatment

Once ICP is diagnosed, 97.3% of participants surveyed start a treatment with UDCA. 39.5% start with an oral dosage of 10–15 mg/kg/d. Adjustments in dosage would be due to subjective maternal symptoms in 28.4% of given answers. 19.2% of the participants estimate the dosage based on serum bile acid concentrations. 77.5% do not recommend a supplementation of vitamin K in pregnancies with ICP (Table 3).

**Table 3:** Medical treatment of ICP

	*N* (%)
Usage of UDCA	
No, never	5 (1.5%)
Yes, in particular	325 (97.3%)
10–15 mg/kg/d	132 (39.5%)
2–4 × tgl. 250 mg	90 (26.9%)
2–4 × tgl. 500 mg	72 (21.6%)
2–4 × tgl. 750 mg	2 (0.6%)
Missing / other dosage	29 (8.8%)
Abstention	4 (1.2%)
Supplementation of vitamin K	
No, never	259 (77.5%)
Yes, for every patient with ICP	17 (5.1%)
In dependence of the PT	45 (13.5%)
Abstention / unclear answer	13 (3.9%)

Data are *n* (% of respondents)

### Delivery timing and management

**Fig. 2:**
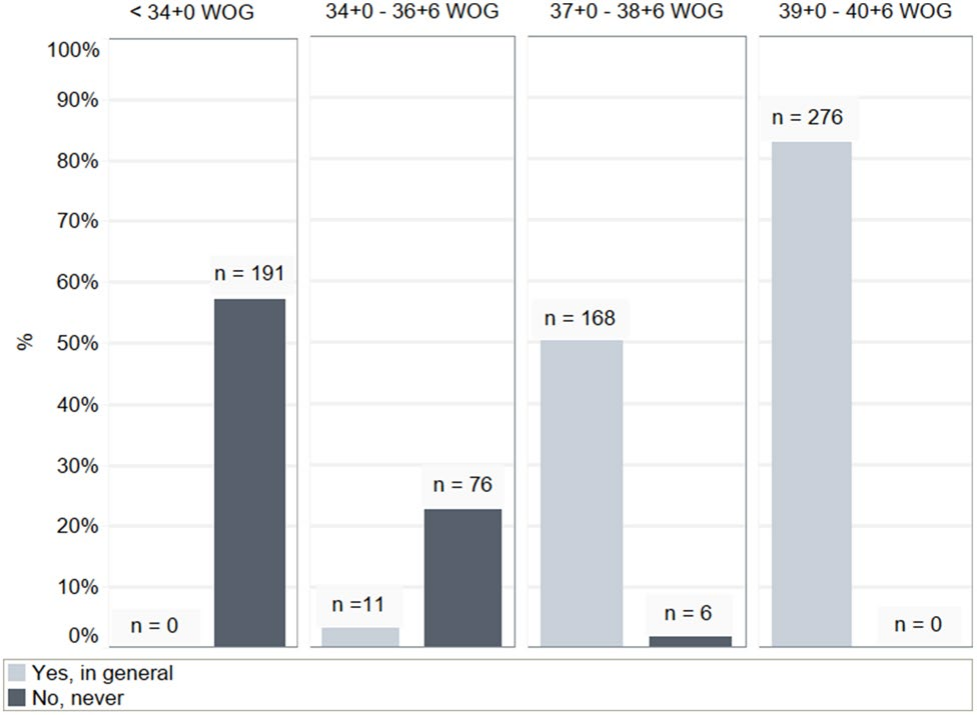
Recommendations for delivery due to ICP in different WOG

**Fig. 3:**
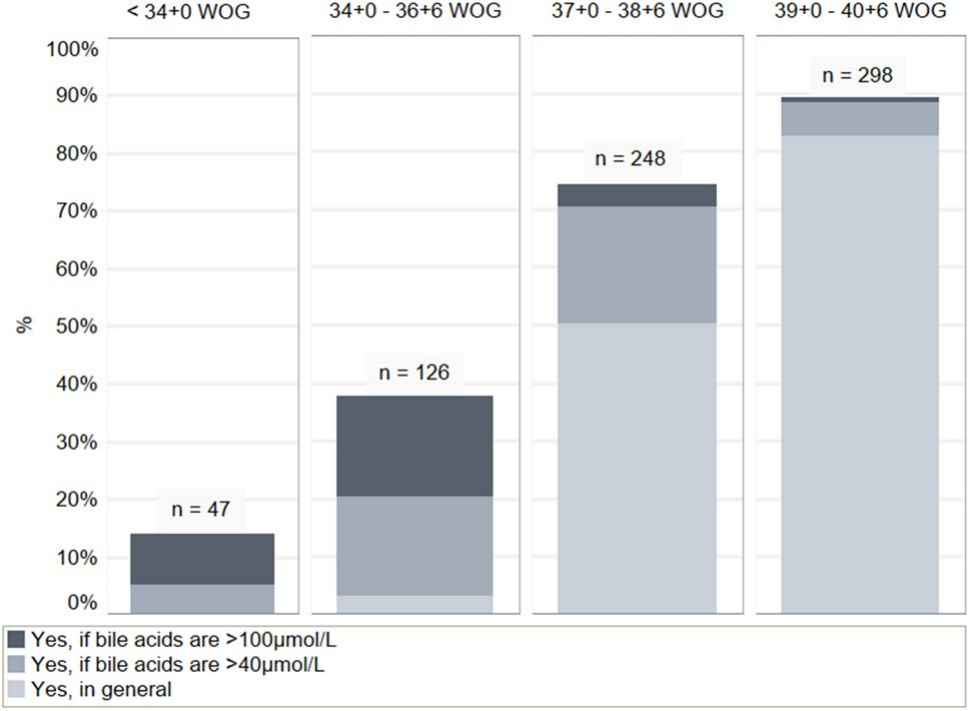
Recommendations for delivery due to raised bile acids in different WOG

Recommendation on induction of labor solely for the presence of ICP (as defined by the participant) was considered in 82.6% between 39 + 0 and 40 + 6 weeks of gestation (WOG), yet 17.4% stated that ICP alone is not sufficient recommending delivery at 39 + 0 WOG. Further details on recommendations for delivery in general are given in Fig. 2. The impact of additional clinical findings (e.g. significant changes in laboratory parameters) on recommendations for delivery are presented in Fig. 3 and Table 4.

**Table 4:** Results of clinical findings and recommendations for delivery consequently in different WOG

	< 34 WOG	34 + 0–36 + 6 WOG	37 + 0–38 + 6 WOG	39 + 0–40 + 6 WOG
Recommendations for delivery in general depending on additional clinical findings				
Delivery in general	0 (0%)	11 (3.3%)	168 (50.3%)	276 (82.6%)
* Plus* significant changes in laboratory parameters	71 (21.3%)	131 (39.2%)	253 (75.7%)	312 (93.4.%)
* Plus* aggravated maternal symptoms despite treatment	30 (9.0%)	80 (24.0%)	250 (74.9%)	319 (95.5%)
* Plus* multiple pregnancies existence	16 (4.8%)	44 (13.2%)	199 (59.6%)	281 (84.1%)

Data are *n* (% of respondents)

### Postpartum care

36.2% of the respondents indicated that they do not recommend postpartum laboratory testing; 42.8% recommend checking on bile acid concentrations and 55.1% repeat LFT after delivery. Nearly one fifth (19.2%) stated that they test coagulations factors postpartum. Nine participants (2.7%) recommend a genetic screening ruling out mutation of bile acids transporters. Most of the participants recommend testing bile acids, LFTs and coagulation factors each within two to six weeks.

## Discussion

In our study, we explored the practice and clinical management of ICP by gynecologists and obstetricians in German maternity and perinatal care units. We demonstrated a heterogenous approach when dealing with ICP. A possible explanation is the scarce evidence and huge differences between international guidelines. So far, an official guideline for treatment and management of ICP in Germany does not exist. Therefore and in the light of the results of this survey, the Working Group on Obstetrics and Prenatal Medicine—Section on Maternal Disorders (AGG) of the German Society of Obstetrics and Gynecology has recently summarized existing evidence on ICP and provides recommendations for the German Health Care System [3]. Evidence based literature supports a clearly defined upper limit of bile acid concentrations in pregnancy [15]. About 30% of study participants deviate from the AGG recommendations and internationally published cut-off values. Moreover, only one third of the participants consider elevated transaminases as indicative for diagnosis. In the presence of pruritus in the setting of ICP, 60% of patients can be expected to have liver enzymes elevations at least two times above normal. The increase in transaminases does not correlate with bile acid levels [3]. Hence, ALT activity may contribute to diagnostic confirmation even in the absence of bile acid elevation. Although the general awareness of the clinical features of ICP was reasonable, the variation of obstetricians’ opinions regarding diagnostic criteria might subsequently lead to significant variations in the management of ICP. Recommendations resulting out of our survey show a predominant role of CTG testing in German maternity hospitals despite its limited or unknown value in the setting of ICP. This goes along with the fact that the CTG is nowadays the most frequently used obstetric measure for assessing fetal well-being predominantly during birth and in pregnancies with increased risk of complications [16, 17]. Despite concerns regarding the reliability and reproducibility of the antenatal CTG as a test of fetal assessment, it has been widely integrated into maternity care practice internationally and especially in German maternity units [17]. The policy of regular fetal monitoring mistakenly suggests a possibility to reduce adverse perinatal outcomes. Although alterations in CTGs were reported, no effective prevention of IUFD could be found in ICP [15]. Fetal death is usually sudden, not predictable [17, 18]. However, gynecologists and obstetricians, fearing legal accusations, may falsely reassure themselves and patients by making clinical examination and observation more intensive. A consensus on the optimal timing of delivery in ICP-affected pregnancies is lacking and poses a controversy. Active management and induction of labor at 37–38 weeks has been recommended by four out of six guidelines reviewed by Bicocca et al. Under the terms of an individual decision-making the current guideline for induction of labor of the Association of the Scientific Medical Societies in Germany (AWMF) addresses ICP and states that a prolongation of the pregnancy beyond 37 + 0 WOG is questionable and ICP-affected women should gave birth at that time [19]. Nonetheless, active management is implemented by only half of the participants responding to the survey. Due to ICP the expective or active management plans are complicated with stillbirth or neonatal preterm morbidities, respectively. A multicenter retrospective cohort study and a systematic meta-analysis have evidently shown that serum bile acid concentrations above 100 µmol/L are associated with an increased risk of stillbirth in women with ICP and singleton pregnancies [20, 21]. The prevalence of stillbirth in the review was three (0.13%; 95% confidence interval (CI) 0.02–0.38) of 2310 ICP cases in women with serum total bile acids of less than 40 µmol/L versus four (0.28%; 0.08–0.72) out of 1412 cases with total bile acids of 40–99 µmol/L (hazard ratio (HR) 2.35 [95% CI 0.52–10.50]; *p* = 0.26), and versus 18 (3.44%; 2.05–5.37) of 524 cases for bile acids of 100 µmol/L or more (HR 30.50 [8.83–105.30]; *p* < 0.0001) [21]. Ovadia et al. indicate that because most women with ICP have concentrations below that level, they can probably be reassured that the risk of stillbirth is similar to the general population, provided repeat bile acid testing is done until delivery [21]. The German AWMF guideline on induction of labor now acknowledges these findings: bile acid levels are a predictive marker for stillbirth and neonatal complications after birth. Hence, delivery at 36 weeks or earlier is solely justifiable due to an individual risk assessment, including severe cases with bile acid concentrations above 100 µmol/L [19]. Three surveys on this issue were conducted in the United Kingdom (UK) in 2006, in France in 2010 and in Australia/New Zealand in 2014 [22–24]. In contrast to our results in Germany, induction of labor at 37 to 38 weeks was a common measure and was reported by 81.3% of Australian up to 92.4% of French survey participants. Coinciding with the results of this survey Saleh & Abdo demonstrate that most British obstetricians perform CTG (84%), ultrasound examinations (82%) and monitor umbilical artery Doppler velocimetry (77%) in ICP-affected pregnancies [22]. The authors of the French study further concluded that the potential of an IUFD is overestimated by their participants, resulting in a high significance of fetal monitoring [23]. Since the studies were carried out years ago and new insights like the significance of bile acids exceeding 100 µmol/L and the risk of an IUFD have been published, it remains unclear how managements of ICP might have changed over the time. Another risk factor for IUFD and unfavorable perinatal outcomes in ICP is multiple pregnancies. Not only is the prevalence of ICP higher in multiple pregnancies [5, 25], ICP also appears to be more severe and stillbirth seems to occur earlier than in singleton pregnancies [5, 26, 27]. However, respondents seem to have little awareness of this, and only a small proportion pay particular attention to multiple pregnancy in their treatment. UDCA is commonly used as off-label in the treatment of ICP [3]. The recent PITCHES trial aimed to evaluate whether UDCA reduces adverse perinatal outcomes in women with ICP [12]. The post-randomization maternal itch score was lower in the UDCA group than in the placebo group (mean difference on a visual analog scale − 5.7 mm [95% CI − 9.7 to − 1.7], *p* = 0·0054); the incidence of the primary outcome (perinatal death, preterm delivery, or neonatal unit admission for ≥ 4 h) did not differ significantly between the UDCA group (23%) and the placebo group (27%) (adjusted RR 0.85 [95% CI 0.62–1.15], p = 0.28) [12]. A Cochrane Review on pharmacological interventions for treating ICP published in 2020, also concluded, when compared with placebo, UDCA probably shows a reduction in pruritus (moderate-certainty evidence) [28]. Although UDCA has not been shown to prevent the adverse outcomes of ICP, it is the only effective treatment with a maternal itching reduction [28]. Despite the potential consequences for affected women due to the risk of an IUFD, on the one hand, and the large number of those referred for treatment to prevent IUFD, on the other hand, few studies have been conducted in this area. ClinicalTrial.gov reports only one interventional trial to evaluate the efficacy and safety of Volixibat, a promising apical sodium-dependent bile acid transporter (ASBT) inhibitor on the course of ICP (NCT04718961). A limitation of our study is that data are based on self-report and therefore cannot be judged objectively. As the survey has been a voluntary approach to the gynecologists and obstetricians, we assume a response bias. The physicians who completed the questionnaire may be more interested in ICP and aware of guidance suggested by evidence-based literature. In addition, we addressed only hospital attending obstetricians as the final decision about management and induction of labor relies on them. Yet, pregnant women in Germany are regularly treated by gynecologists in the outpatient setting. The dual care system of out- and inpatient settings in addition to inadequate cooperation can also lead to a delay in referral to the hospital. Therefore, future surveys must include gynecologists who practice medicine only in the outpatient setting. Our survey with regard the diagnosis, care, and treatment of pregnant women with ICP is the first of its kind. A major strength is a robust and easy fillable questionnaire, allowing all clinically important aspects to be addressed. This may have led to a high response rate.

## Conclusion

The management of ICP in Germany is heterogenous and does not correspond to the international standards. Current aspects of evidence-based studies are not considered in the management by large. It includes the significance of bile acids exceeding 100 µmol/L and the risk of an IUFD for example. Furthermore, it shows the predominant role of CTG testing in German maternity hospitals despite the lack of evidence. Due to an apparent necessity of a national consensus, the Working Group on Obstetrics and Prenatal Medicine—Section on Maternal Disorders (AGG) of the German Society of Obstetrics and Gynecology has recently developed recommendations on the treatment of ICP for German Health Care System [3]. Our survey therefore highlights the importance of these recommendations to standardize German practices with regard to ICP.

## Supplementary Information

Below is the link to the electronic supplementary material.

Supplementary file1 (PDF 204 KB)
