# Supplementary material for: Intrahepatic cholestasis of pregnancy: an evaluation of obstetric management in German maternity units
Source: Arch Gynecol Obstet. 2022 Aug 28;308(3):831–8. doi: 10.1007/s00404-022-06754-3 (PMC10349004; doi:10.1007/s00404-022-06754-3)
Supplement: Supplementary file 1 — Supplementary file1 (PDF 204 KB) [file 404_2022_6754_MOESM1_ESM.pdf]

Intrahepatic cholestasis of pregnancy: an evaluation of obstetric management in German maternity units

Leonie Zehner[1] · Maria Mai[1] · Anna M Dückelmann[2] · Amr Hamza[3] · Christel Eckmann-Scholz[1,4] · Nicolai Maass[1,4] · Ulrich Pecks[1,4]

✉ Prof. Dr. med. Ulrich Pecks
Ulrich.Pecks@uksh.de

[1] Medical Faculty
Christian-Albrechts-Universität Kiel,
Christian-Albrechts-Platz 4,
24118 Kiel, Germany

[4] Department of Gynaecology and Obstetrics
Universitätsklinikum Schleswig-Holstein,
Arnold-Heller-Straße 3,
24105 Kiel, Germany

A questionnaire focusing on the treatment and management of ICP

1. Is a clinical guideline principle / a written concept for management of ICP existing in the unit?

- Yes.
- No.

---

2. Which laboratory parameters are the main criteria for diagnosis of ICP?/Which laboratory parameters should be fulfilled mandatorily confirming the diagnosis of ICP? (multiple responses allowed)

- Pruritus (generalized or on the palms/soles respectively)
- Jaundice
- Elevated bilirubin
- Elevated liver enzymes
- Elevated γGT
- Elevated bile acid concentrations

---

3. Should the fasting status of bile acids be considered in the determination?

- Yes, bile acids should be tested with fasting patients.
- No, the fasting status is insignificant.

---

4. Which score of serum bile acid concentration ensures the diagnosis of ICP?

- None. The bile acid concentration does not play a role for diagnosis.
- Following cut-off defines diagnosis:
    - > 10µmol/L
    - > 15µmol/L
    - > 40µmol/L
    - > 100µmol/L

---

5. Is laboratory testing recommended as a matter of routine?

- No.
- Yes, in particular:

  daily   every 2 to 3 days   every 4 to 6 days   every 7 to 13 days   > 14 days

---

6. Is fetal monitoring advised throughout an ICP-affected pregnancy? (multiple responses allowed)

- No specific monitoring.
- CTG:
  daily   every 2 to 3 days   weekly   depending on symptoms and laboratory parameters
- Doppler:
  daily   every 2 to 3 days   weekly   depending on symptoms and laboratory parameters
- Sonography:
  daily   every 2 to 3 days   weekly   depending on symptoms and laboratory parameters

---

7. Does the participant recommend the use of UDCA?

- No.
- Yes, in particular: 10-15mg/kg/d   2-4x 250mg/d   2-4x 500mg/d   2-4x 750mg/d

---

8. What is the most important parameter which adjusts the dosage of UDCA?

- I recommend prescribing a standard dosage.
- Dosage adapts to maternal psychological strains / extent of maternal symptoms (pruritus).
- Dosage adapts to the extent of transaminases levels.
- Dosage adapts to the bile acid concentration.

---

9. Should Vitamin K be prescribed in cases of ICP?

- No.
- In all patients with ICP.
- If the prothrombin time is prolonged.

---

10. Does the participant recommend a delivery before 34+0 weeks of gestations (WOG) due to ICP and under certain circumstances? (multiple responses allowed)

- No, never. I advise against a delivery in this WOG.
- Yes, in general. I recommend delivery in this WOG.
- Yes, in general if laboratory parameters change significantly.
- Yes, in general if maternal symptoms aggravate despite treatment.
- Yes, in general if ICP occurs in multiple pregnancies.
- Yes, in general if bile acid concentrations are elevated. If bile acids are
  - > 40µmol/L
  - > 100µmol/L

---

11. Does the participant recommend a delivery between 34+0 and 36+6 WOG due to ICP and under certain circumstances? (multiple responses allowed)

- No, never. I advise against a delivery in this WOG.
- Yes, in general. I recommend delivery in this WOG.
- Yes, in general if laboratory parameters change significantly.
- Yes, in general if maternal symptoms aggravate despite treatment.
- Yes, in general if ICP occurs in multiple pregnancies.
- Yes, in general if bile acid concentrations are elevated. If bile acids are
  - > 40µmol/L
  - > 100µmol/L

---

12. Does the participant recommend a delivery between 37+0 and 38+6 WOG due to ICP and under certain circumstances? (multiple responses allowed)

- No, never. I advise against a delivery in this WOG.
- Yes, in general. I recommend delivery in this WOG.
- Yes, in general if laboratory parameters change significantly.
- Yes, in general if maternal symptoms aggravate despite treatment.
- Yes, in general if ICP occurs in multiple pregnancies.
- Yes, in general if bile acid concentrations are elevated. If bile acids are
  - > 40µmol/L
  - > 100µmol/L

---

13. Does the participant recommend a delivery between 39+0 and 40+6 WOG due to ICP and under certain circumstances? (multiple responses allowed)

- No, never. I advise against a delivery in this WOG.
- Yes, in general. I recommend delivery in this WOG.
- Yes, in general if laboratory parameters change significantly.
- Yes, in general if maternal symptoms aggravate despite treatment.
- Yes, in general if ICP occurs in multiple pregnancies.
- Yes, in general if bile acid concentrations are elevated. If bile acids are
  - > 40µmol/L
  - > 100µmol/L

---

14. Are monitoring and controls recommended postpartum? (multiple responses allowed)

- o No.
- o Yes, control of bile acid in ________ weeks.
- o Yes, control of transaminases in ________ weeks .
- o Yes, control of blood coagulation factors in ________ weeks.
- o Genetic examinations (excluding mutations of transporters e.g.).

Demographic and personal data

First number of postcode: ___________ Age: __________ Sex: ______

Medical specialization:	Obstetrics and gynecology
	Obstetrics and perinatal medicine
	Trainees in obstetrics and gynecology
Status of employment:	Resident physician
	Consultant physician
	Senior physician
	Senior consultant physician
	Head of department
Working place:	Maternity clinic
	Perinatal care center level I
	Perinatal care center level II
	Perinatal care center level III
Birth per year:	< 1000
	1000 – 1999
	2000 – 3000
	> 3000
